# Pre-implementation adaptation of primary care cancer prevention clinical decision support in a predominantly rural healthcare system

**DOI:** 10.1186/s12911-020-01136-8

**Published:** 2020-06-23

**Authors:** Melissa L. Harry, Daniel M. Saman, Anjali R. Truitt, Clayton I. Allen, Kayla M. Walton, Patrick J. O’Connor, Heidi L. Ekstrom, JoAnn M. Sperl-Hillen, Joseph A. Bianco, Thomas E. Elliott

**Affiliations:** 1 Essentia Health, Essentia Institute of Rural Health, 6AV-2, 502 East Second Street, Duluth, MN 55805 USA; 2 HealthPartners Institute, 3311 E. Old Shakopee Road, Bloomington, MN 55425 USA; 3 Essentia Health – Ely Clinic, 300 W Conan Street, Ely, MN 55731 USA

**Keywords:** Cancer prevention and screening, Clinical decision support, Consolidated Framework for Implementation Research, Pre-implementation adaptation, Primary care, Shared decision-making tools

## Abstract

**Background:**

Cancer is a leading cause of death in the United States. Primary care providers (PCPs) juggle patient cancer prevention and screening along with managing acute and chronic health problems. However, clinical decision support (CDS) may assist PCPs in addressing patients’ cancer prevention and screening needs during short clinic visits. In this paper, we describe pre-implementation study design and cancer screening and prevention CDS changes made to maximize utilization and better fit a healthcare system’s goals and culture. We employed the Consolidated Framework for Implementation Research (CFIR), useful for evaluating the implementation of CDS interventions in primary care settings, in understanding barriers and facilitators that led to those changes.

**Methods:**

In a three-arm, pragmatic, 36 clinic cluster-randomized control trial, we integrated cancer screening and prevention CDS and shared decision-making tools (SDMT) into an existing electronic medical record-linked cardiovascular risk management CDS system. The integrated CDS is currently being tested within a predominately rural upper Midwestern healthcare system. Prior to CDS implementation, we catalogued pre-implementation changes made from 2016 to 2018 based on: pre-implementation site engagement; key informant interviews with healthcare system rooming staff, providers, and leadership; and pilot testing. We identified influential barriers, facilitators, and changes made in response through qualitative content analysis of meeting minutes and supportive documents. We then coded pre-implementation changes made and associated barriers and facilitators using the CFIR.

**Results:**

Based on our findings from system-wide pre-implementation engagement, pilot testing, and key informant interviews, we made changes to accommodate the needs of the healthcare system based on barriers and facilitators that fell within the *Intervention Characteristics*, *Inner Setting*, and *Outer Setting* CFIR domains. Changes included replacing the expansion of medical assistant roles in one intervention arm with targeted SDMT, as well as altering cancer prevention CDS and study design elements.

**Conclusions:**

Pre-implementation changes to CDS may help meet healthcare systems’ evolving needs and optimize the intervention by being responsive to real-world implementation barriers and facilitators. Frameworks like the CFIR are useful tools for identifying areas where pre-implementation barriers and facilitators may result in design changes, both to research studies and CDS systems.

**Trial registration:**

NCT02986230.

## Background

A Healthy People 2020 goal aims to reduce cancer incidence, morbidity, and mortality, including through supporting evidence-based recommendations made by the U.S. Preventive Services Task Force (USPSTF), which regularly updates prevention recommendations for common cancers [1]. This is particularly relevant in primary care, where patients are most likely to receive a variety of cancer prevention recommendations [2]. However, some common cancer screening tests can pose health risks to patients [3], risks that may not be adequately explained by providers [4–6].

Shared decision-making between patients and providers is a key component of patient-centered care, particularly for medical decisions with multiple options bearing varied risks [7]. Unlike the paternalistic medical model where physicians make medical decisions for patients [8, 9], shared decision-making aims to help patients make informed choices about their health with the assistance of their provider and the latest information that fits their lifestyle and personal preferences [3].

Clinical decision support (CDS) systems can be used to inform patients of potential benefits and risks of evidence-based treatment options, providing decision support as envisioned in the Chronic Care Model [10]. CDS systems typically draw information from patients’ electronic health records (EHR), process the information using algorithms employing evidence-based guidelines, then present tailored clinical recommendations to providers and patients [11, 12]. For example, CDS systems can identify patients eligible for cancer prevention and screening. They can also provide decision aids, such as shared decision-making tools (SDMT). Research has shown that decision aids enhance shared decision-making between patients and providers, and boost patient knowledge of treatment options and associated risks [13]. A review of cancer screening and treatment decision aids showed patients may increase their knowledge and make more informed, personally-tailored choices when using decision aids [14]. Typical cancer prevention SDMT feature an introduction, risks and benefits of available screening modalities, supplementary information like risk calculators, and statements or questions aimed at helping patients decide among available options [15].

Other innovative service delivery methods may also facilitate shared decision-making. Considering the impending primary care physician shortage [16], including due to burnout [17, 18], research illustrates the benefits of taking some of the load off physicians by utilizing medical assistants in expanded roles [17]. While few states allow medical assistants to provide medical advice, assessment, or triage patient problems, they can perform specific physician-supervised duties when sufficiently trained [18]. This model has been successfully implemented into practice and evaluated within some healthcare centers [19, 20]. However, few randomized control trials with sufficient power have been conducted [21–23], including trials examining the generalizability of expanding medical assistant roles in addressing patients’ primary and secondary cancer prevention and screening needs in rural clinic settings, which can experience a dearth of providers and other critical resources [24].

### Purpose and guiding research question

Dissemination and implementation (D&I) science can aid in identifying both barriers and facilitators to the adoption and usage (implementation), as well as spread (dissemination) of interventions [25]. Our study adds to this body of knowledge by providing an example of employing the Consolidated Framework for Implementation Research (CFIR) [26] in understanding pre-implementation study protocol and intervention changes made based on barriers and facilitators encountered within a predominately rural, multi-state healthcare system. Changes were made to fit institutional preferences and aid implementation and adoption of a cancer prevention and screening CDS in a pragmatic trial currently being conducted in a real-world environment. While other literature focuses on adapting existing interventions, the literature is scarce on studies that examine adaptations made based on barriers and facilitators encountered when upgrading or enhancing current CDS systems. To help fill that gap, this paper was guided by the following research question: 1) How did the study team adapt the study design and the cancer prevention CDS to meet institutional needs, culture, and goals prior to implementation?

## Methods

### Our study’s original design and evaluation setting

We originally planned on studying a model of trained medical assistants providing scripted information generated by a cancer prevention and screening CDS system to patients in need of primary and secondary cancer prevention to enhance shared decision-making and improve cancer prevention and screening rates in a largely rural primary care population. Our approved National Institutes of Health grant application outlined developing and testing an EHR-linked, web-based, point-of-care cancer prevention CDS system that addressed both primary (obesity, smoking, HPV vaccination) and secondary (breast, cervical, colorectal) cancer prevention through a three-arm clinic cluster-randomized control trial in a multi-state integrated healthcare system. Specifically, randomization occurred at the clinic level. The healthcare system has over 60 primary care clinics and 13 hospitals in the upper Midwest and serves a largely rural patient population. Primary care clinics in this study are located in Minnesota, North Dakota, and Wisconsin. Original study arms included a control arm receiving usual care (current cancer screening and prevention practices at the primary care clinic-level) and two intervention arms: one arm with primary care providers (PCPs) using the cancer prevention CDS (also known as the Priority Wizard) with patients after it is printed and distributed by clinic rooming staff (including medical assistants), and another arm with trained medical assistants providing scripted cancer prevention CDS recommendations and beginning relevant orders for eligible patients prior to the PCP entering the room and completing the shared decision-making discussion with the patient. Both intervention arms would receive separate printed PCP and patient interface handouts outlining and prioritizing personalized items for primary and secondary cancer prevention and screening. While healthcare system leadership initially believed this study design feasible, pre-implementation engagement with broader members of the multi-state healthcare system showed otherwise. Consequently, the study team made considerable pre-implementation changes from the start of the study (April 2016) through implementation (June 2018). These modifications altered the CDS design by incorporating it into an existing cardiovascular risk management CDS system used in the healthcare system as part of two other National Institutes of Health-funded studies targeting patients with prediabetes/diabetes or serious mental illness. Pre-implementation engagement also resulted in our altering the scope of one intervention arm to include SDMT rather than trained medical assistants (medical assistants and other clinic rooming staff are still responsible for printing and distributing patient and PCP CDS handouts in both intervention arms). The healthcare system’s institutional review board approved these changes and they were reported to the National Institutes of Health program officer without issue.

### Theoretical framework

The CFIR combines 19 implementation-related models into a single meta-framework with five separate domains: *Intervention Characteristics*, *Outer Setting*, *Inner Setting*, *Characteristics of Individuals*, and *Process* [26]. The CFIR can help: evaluate multiple factors affecting the implementation and dissemination of Chronic Care Model interventions [26]; assess pre-implementation activities [26]; and understand pre-implementation changes made based on barriers and facilitators encountered [27, 28]. The CFIR also allows for flexibility to only apply relevant domains and constructs to study data, rather than the entire CFIR [26].

### Data collection

In answering our primary research question, we collected pre-implementation data from three sources: research team and site engagement meeting minutes; key informant interviews [15]; and CDS pilot testing. Meeting minutes were taken by team members from April 2016, the start of the study, through the completion of pilot testing at the end of May 2018. Meetings included: numerous weekly, monthly, quarterly, and annual team meetings, as well as clinic site visits and other ancillary meetings, including with: providers from colorectal cancer screening and oncology; information services; primary care leadership; quality experts; and other subject matter advisors employed by the healthcare system. Meetings typically lasted about one hour. Team leaders also presented at a quarterly primary care leadership meeting where questions were fielded to preempt problems that could turn into implementation or use barriers as able. We also attended a pilot clinic primary care PCP section meeting (i.e., department meeting), where we presented on the cancer prevention CDS and SDMT under development, which influenced their design. All meeting minutes followed the same format, including listing meeting attendees, agenda items, and details on items discussed. Furthermore, between June and September 2017 MLH, CIA, and another study team member interviewed 28 key informants in either leadership roles in the healthcare system or who were PCPs or rooming staff in current cardiovascular CDS intervention clinics [15]. We made some changes to the study based on the results of these interviews [15]. Data saturation, when no new themes emerge from the data, was reached with this sample [15]. Results from that study showed barriers and facilitators relating to the EHR, the CDS workflow, CDS users (PCPs and patients), available training methods for clinics spread out across three predominantly rural states, and the healthcare organization [15]. These barriers and facilitators were then mapped to CFIR domains and constructs [15, 26]. Finally, we conducted two pilots to test CDS algorithms and workflow: first, a six-month silent pilot in one non-study healthcare system clinic where the CDS ran in the background; then we conducted a seven-week live pilot in two additional non-study healthcare system clinics where rooming staff were instructed to print the integrated CDS and SDMT following the recommended workflow. Manual firing of the CDS was also made available to live pilot clinic PCPs. The live pilot clinics were the same clinics that had piloted the cardiovascular CDS at the healthcare system and continued to use the cardiovascular CDS until the integrated CDS that included cancer prevention and screening went live.

### Data analysis

TEE and MLH identified and documented changes made to the study protocol and intervention prior to implementation, including details about what lead to each change. Next, MLH and ART reviewed 121 meeting minute documents to confirm these changes. Lastly, MLH and ART documented the sources of each change, whether the changes represented implementation and adoption barriers or facilitators, coded changes made using CFIR *Intervention Characteristics*, *Inner Setting*, and *Outer Setting* domains [26], as not all domains or constructs applied, and agreed on coding.

## Results

**Fig. 1:**
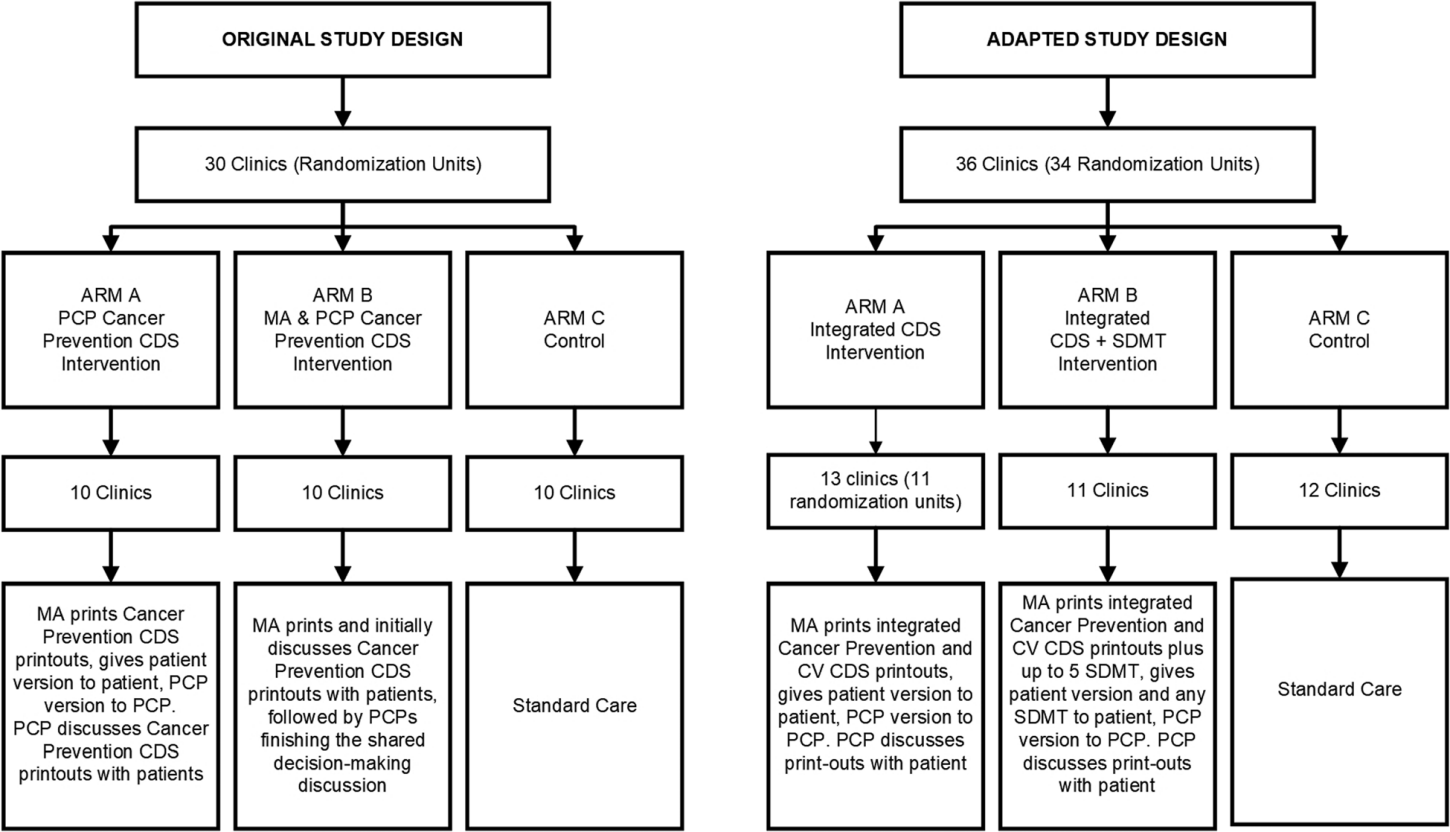
Original and adapted study designs. CDS: Clinical decision support. CV: Cardiovascular. MA: Medical assistant. PCP: Primary care provider. SDMT: Shared decision-making tools

**Fig. 2:**
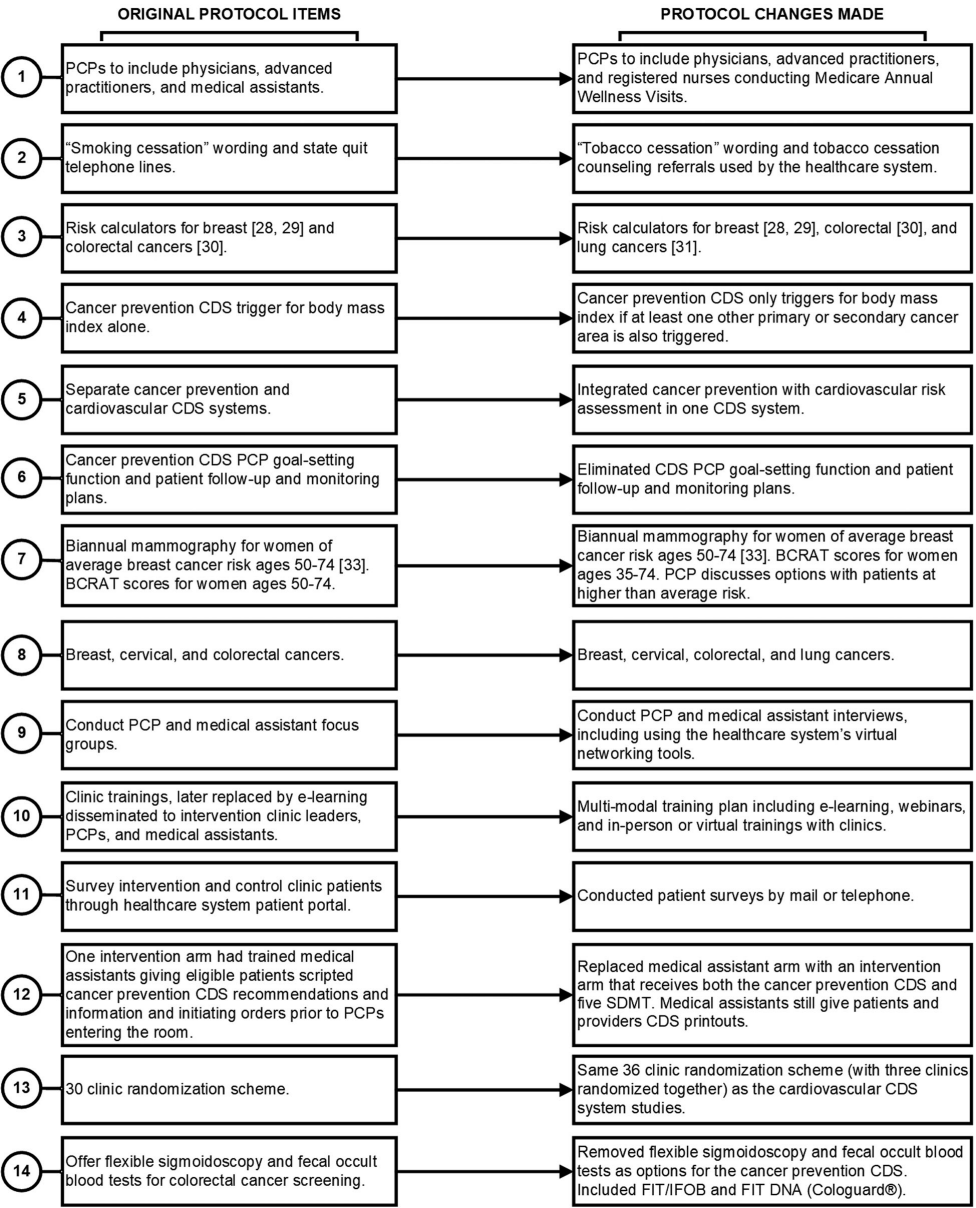
Original and revised protocol items. BCRAT: Breast cancer risk assessment tool. CDS: Clinical decision support. FIT: Fecal immunochemical test. FIT DNA: Fecal immunochemical test deoxyribonucleic acid. IFOB: Immunoassay fecal occult blood test. PCP: Primary care provider. SDMT: Shared decision-making tool

Tables 1 and 2 include CFIR domains and constructs [26], original protocol and intervention components, pre-implementation barriers and facilitators encountered, changes made, and their source (pre-implementation engagement, key informant interviews [15], or pilot testing). Figure 1 depicts the primary original study design alongside the adapted version, focusing on intervention arm and clinic randomization design changes. Figure 2 displays all changes made with numbers that connect to those presented in Tables 1 and 2. We present more detailed description of our results below categorized by CFIR domain and relevant construct(s).

**Table 1:** Study Protocol and Cancer Prevention CDS Changes Made by CFIR Intervention Characteristics Domain and Constructs [26]

CFIR Domain & Related Constructs	Change Number	Elements of the Initial Protocol	Barriers Encountered	Facilitators Encountered	New Protocol (Change Made)	Source of Barriers or Facilitators Encountered
**I. INTERVENTION CHARACTERISTICS**						
D. Adaptability	*The degree to which an intervention can be adapted, tailored, refined, or reinvented to meet local needs.*					
	1	Cancer prevention CDS providers to include PCP physicians, advanced practitioners, and medical assistants.	Intervention arm with medical assistants replaced by an arm where PCPs receive SDMT for eligible patients. Medical assistants no longer considered as study primary care providers.	The cardiovascular CDS system was being triggered for registered nurses conducting Medicare Annual Wellness Visits.	Cancer prevention CDS PCPs include registered nurses conducting Medicare Annual Wellness Visits in both intervention arms. Along with other rooming staff, medical assistants continue to receive the best practice alert that triggers printing of the cancer prevention CDS in both the integrated CDS and integrated CDS + SDMT intervention arms.	Pre-implementation engagement (Healthcare system cardiovascular CDS team members). Key informant interviews.
	2	Cancer prevention CDS used the phrase “smoking cessation” and state quit telephone lines.	Healthcare system refers to “tobacco cessation”.	The healthcare system had recently initiated a tobacco cessation counseling program.	Rephrased to use tobacco cessation wording and tobacco cessation counseling referrals used by the healthcare system.	Pre-implementation engagement (Healthcare system primary care leaders and technology learning and support staff). Key informant interviews.
	3	Risk calculators for breast [29, 30] and colorectal cancers [31].		Healthcare system offered lung cancer screening. Healthcare system leadership asked for a lung cancer risk calculator to be included.	Added a lung cancer risk calculator [32].	Pre-implementation engagement (Healthcare system primary care and other leaders).
E. Trialability	*The ability to test the intervention on a small scale in the organization, and to be able to reverse course (undo implementation) if warranted.*					
	4	Cancer prevention CDS trigger for body mass index alone.	Pilot testing showed frequent triggering of the cancer prevention CDS based on body mass index alone.	Healthcare system-level goal to address body mass index. Body mass index also triggers the cardiovascular CDS.	The cancer prevention CDS only triggers for body mass index if at least one other primary or secondary cancer area is also triggered.	Pre-implementation engagement (Healthcare system primary care leaders). Pilot testing.
F. Complexity	*Perceived difficulty of implementation, reflected by duration, scope, radicalness, disruptiveness, centrality, and intricacy and number of steps required to implement.*					
	5	Separate cancer prevention and cardiovascular CDS systems.	Potential burden of two separate CDS systems on PCPs.	One institution is designing both CDS systems. Preference for a single system by healthcare system leadership. Different implementation and intervention dates.	Integrating cancer prevention with cardiovascular risk assessment in one CDS system.	Pre-implementation engagement (Healthcare system primary care and other leaders).
	6	Cancer prevention CDS PCP goal-setting function and patient follow-up and monitoring plan.	Inconsistent patient follow-up and monitoring infrastructure across the healthcare system’s three markets.	Healthcare system has own system of best practice advisories and screening and prevention recommendations within the EHR.	Eliminated CDS goal-setting function and patient follow-up and monitoring plan.	Pre-implementation engagement (Healthcare system staff managing patient communication).

*Note.* Only CFIR [26] domains and constructs relevant to study changes are included here. Complete key informant interview results are reported elsewhere [15]. CDS, clinical decision support; EHR, electronic health record; PCP, primary care provider; SDMT, shared decision-making tool

**Table 2:** Study Protocol and Cancer Prevention CDS Changes Made by CFIR Outer Setting and Inner Setting Domains and Constructs [26]

CFIR Domains & Related Constructs	Change Number	Elements of the Initial Protocol	Barriers Encountered	Facilitators Encountered	New Protocol (Change Made)	Source of Barriers or Facilitators Encountered
**II. OUTER SETTING**						
D. External Policy & Incentives	*A broad construct that includes external strategies to spread interventions, including policy and regulations (governmental or other central entity), external mandates, recommendations and guidelines, pay-for-performance, collaboratives, and public or benchmark reporting.*					
	7	Biannual mammography for women of average breast cancer risk ages 50 to 74 based on USPSTF guidelines [34]. Also providing BCRAT scores for women ages 50 to 74.	Healthcare system encouraging annual mammography starting at age 40 for all women, which does not align with USPSTF guidelines [34] followed in the cancer prevention CDS.		Offer biannual mammography for women of average breast cancer risk ages 50 to 74 based on USPSTF guidelines [34]. Recommend discussion with PCP for women at higher than average risk ages 35 to 49 due to the BCRAT calculating scores from ages 35 and up [29, 30].	Pre-implementation engagement (primary care leaders).
	8	Targeted secondary cancer screenings: breast, cervical, and colorectal cancers.		Healthcare system leadership asked for lung cancer screening to be included to encompass all four USPSTF recommended screenings [33–36]. Healthcare system offered lung cancer screening.	Added lung cancer screening to the cancer prevention CDS.	Pre-implementation engagement (Healthcare system primary care and other leaders).
**III. INNER SETTING**						
B. Networks & Communications	*The nature and quality of webs of social networks and the nature and quality of formal and informal communications within an organization.*					
	9	Conduct PCP and medical assistant focus groups.	Healthcare system study clinics span three upper Midwestern states in predominately rural areas.	Healthcare system has virtual networking capabilities and tools.	Conduct PCP and medical assistant interviews, including using the healthcare system’s virtual networking tools.	Pre-implementation engagement (Healthcare system primary care leaders).
	10	Clinic trainings, later replaced by e-learning disseminated to intervention clinic leaders, PCPs, and medical assistants.	Uneven uptake of the previous cardiovascular CDS system's e-learning.	Recommendations for multiple learning points and training types.	Multi-modal training plan including e-learning, webinars recorded and uploaded to intranet, and in-person/virtual trainings with clinics over a 6-month post-implementation window.	Key informant interviews.
	11	Surveying intervention and control clinic patients through the healthcare system's patient portal.	All patient surveys must first go through the healthcare system’s marketing department. Patient surveys cannot be targeted to specific clinics through the patient portal.	Institution developing the cancer prevention CDS has a Survey Research Center.	Using the Survey Research Center to survey study patients either through: written mailed or telephone surveys.	Pre-implementation engagement (Healthcare system marketing and patient portal departments).
D. Implementation Climate	*The absorptive capacity for change, shared receptivity of involved individuals to an intervention, and the extent to which use of that intervention will be rewarded, supported, and expected within their organization.*					
2. Compatibility	*The degree of tangible fit between meaning and values attached to the intervention by involved individuals, how those align with individuals’ own norms, values, and perceived risks and needs, and how the intervention fits with existing workflows and systems.*					
	12	One intervention arm to have trained medical assistants give eligible patients scripted cancer prevention CDS recommendations and information, as well as initiate orders, prior to PCPs entering the room.	Medical assistants cannot initiate all necessary orders for PCPS in the healthcare system EHR. Medical assistants do not have similar roles in the cardiovascular CDS system studies. Decision to have only one integrated CDS system with both cancer prevention and cardiovascular risk reduction goals.	Healthcare system interest in shared decision-making and SDMT. Medical assistants already asked to print and distribute cardiovascular CDS materials for patients and PCPs.	Replaced medical assistant arm with an intervention arm that receives both the cancer prevention CDS and five SDMT. Medical assistants, and other rooming staff, are still trained to provide patient cancer prevention CDS materials to patients prior to PCPs entering the room and give PCPs’ print outs to PCPs in both intervention arms.	Pre-implementation engagement (Healthcare system primary care leaders, gastroenterology shared decision-making group, breast cancer shared decision-making group, technology learning and support staff, and cardiovascular CDS study staff).
	4	Cancer prevention CDS trigger for body mass index alone.	Pilot testing showed frequent triggering of the cancer prevention CDS based on body mass index alone.	Healthcare system-level goal to address body mass index. Body mass index also triggers the cardiovascular CDS.	The cancer prevention CDS only triggers for body mass index if at least one other primary or secondary cancer area is also triggered.	Pre-implementation engagement (Healthcare system primary care leaders). Pilot testing.
	6	Cancer prevention CDS PCP goal-setting function and patient follow-up and monitoring plan.	Inconsistent patient follow-up and monitoring infrastructure across the healthcare system’s three markets.	Healthcare system has own system of best practice advisories and screening and prevention recommendations within the EHR.	Eliminated CDS PCP goal-setting function and patient follow-up and monitoring plan.	Pre-implementation engagement (Healthcare system staff managing patient communication).
E. Readiness for Implementation	*Tangible and immediate indicators of organizational commitment to its decision to implement an intervention.*					
1. Leadership Engagement	*Commitment, involvement, and accountability of leaders and managers with the implementation.*					
	3	Risk calculators for breast [29, 30] and colorectal cancers [31].		Healthcare system offered lung cancer screening. Healthcare system leadership asked for a lung cancer risk calculator to be included.	Added a lung cancer risk calculator [32].	Pre-implementation engagement (Healthcare system primary care and other leaders).
2. Available Resources	*The level of resources dedicated for implementation and on-going operations, including money, training, education, physical space, and time.*					
	13	30 clinic randomization scheme.	The cardiovascular CDS system studies already included the largest healthcare system clinics.	36 clinic randomization scheme (with three clinics randomized together) for the cardiovascular CDS system studies.	Same 36 clinic randomization scheme (with three clinics randomized together) as the cardiovascular CDS system studies.	Pre-implementation engagement (Healthcare system primary care leaders and cardiovascular CDS study team).
	14	Offer flexible sigmoidoscopy and fecal occult blood tests for colorectal cancer screening.	The healthcare system no longer offers flexible sigmoidoscopy or fecal occult blood tests.	The healthcare system offers FIT (also referred to as IFOB) and FIT Cologuard® DNA tests, as well as colonoscopy.	Removed flexible sigmoidoscopy and fecal occult blood tests as options for the cancer prevention CDS. Included FIT/IFOB and FIT DNA (Cologuard®) options.	Pre-implementation engagement (Healthcare system primary care leaders, gastroenterology department members, and EHR programmers).

*Note.* Only CFIR [26] domains and constructs relevant to study changes are included here. Complete key informant interview results are reported elsewhere [15]. CDS, clinical decision support; D&I, dissemination and implementation; EHR, electronic health record; FIT/FIT DNA, fecal immunochemical test deoxyribonucleic acid; IFOB, immunoassay fecal occult blood test; PCP, primary care provider; SDMT, shared decision-making tool; USPSTF, U.S. Preventative Services Task Force

### I. Intervention characteristics (Table 1)

#### D. Adaptability

We added registered nurses conducting Medicare Annual Wellness Visits as PCPs, as the cardiovascular CDS system triggered for these nurses and many used it. Primary care leadership and registered nurses also gave positive feedback on this role continuing with the cancer prevention CDS. “Tobacco” replaced “smoking” terminology to incorporate chewing products and healthcare system tobacco cessation counseling. Furthermore, in addition to risk calculators for breast and colorectal cancers [29–31], we added a lung cancer risk calculator [32], as healthcare system leaders asked for one to be included and the healthcare system had in place low-dose CT scans, policies, and procedures to ensure appropriate use of this intervention (also coded III. Inner Setting, E. Readiness for implementation, 1. Leadership engagement. See Table 2) [26]. Healthcare system leadership had asked our team to study all four types of cancer with USPSTF screening recommendations (lung, breast, colorectal, cervical) [33–36] (coded II. Outer Setting, D. External policy & incentives. See Table 2) [26].

#### E. Trialability

Pilot testing lead to our reducing the frequency that patient body mass index triggers the cancer prevention CDS; it now only triggers along with at least one cancer prevention-related area. The cardiovascular CDS still triggers for body mass index alone for patients with prediabetes/diabetes or serious mental illness (also coded III. Inner Setting, D. Implementation Climate, 2. Compatibility. See Table 2) [26].

#### F. Complexity

All three research teams and healthcare system primary care leadership decided to integrate cancer prevention with the cardiovascular CDS into a single, unified CDS system to avoid overburdening PCPs and rooming staff. Prediabetes/diabetes and serious mental illness patients can also benefit from cancer screening and prevention. Furthermore, these studies are pragmatic trials conducted in real-world settings where dealing with both cardiovascular risk and cancer prevention is routine and changing guidelines and other interventions are the norm. Moreover, both cardiovascular CDS studies had already been collecting data for over a year prior to the addition of cancer prevention CDS. Study research questions did not change based upon this integration, as all focus on separate patient outcomes.

We originally included patient follow-up and monitoring plans and a PCP goal-setting function in the cancer prevention CDS. We eliminated these plans due to the incomplete system-wide infrastructure for PCP goal-setting and patient follow-up and monitoring across the three healthcare system markets (also coded III. Inner Setting, D. Implementation Climate, 2. Compatibility. See Table 2). The cancer prevention CDS triggered once every 120 days for eligible patients during the intervention period, once every two weeks for eligible patients in the cardiovascular CDS studies, and now triggers once every two weeks for all eligible patients during the 12-month intervention follow-up, helping keep tabs on patients not up-to-date.

### II. Outer setting (Table 2)

#### D. External policy & incentives

The healthcare system recommends annual mammography for all women aged 40 and over. However, the cancer prevention CDS follows USPSTF recommendations of biannual mammography for average-risk women ages 50 to 74 [34]. For women ages 35 to 49 identified as being at higher-than-average risk [29, 30], the cancer prevention CDS recommends PCPs speak with their patient about options related to increased breast cancer risk. As noted unter I. Intervention Characteristics, D. Adaptability, at the request of healthcare system leadership, we added lung cancer screening in order to encompass all four types of cancer with USPSTF screening recommendations [33–36]. 

### III. Inner setting (Table 2)

#### B. Networks & communications

Due to the far-flung and rural nature of the healthcare system with study clinics across three upper Midwestern U.S. states, we determined conducting post-implementation PCP and medical assistant interviews using the healthcare system’s virtual networking capabilities would be more feasible than the original focus group plans. Individual clinic trainings were initially supplanted by the cardiovascular CDS training model that relied on top-down dissemination of e-learning and standard workflow documents from clinic managers to PCPs and rooming staff. However, key informant interviews of cardiovascular CDS users showed inconsistent uptake of this training model [15]. Based on key informant recommendations [15], we developed a multi-modal training plan including e-learning webinars and in-person clinic trainings. Using the healthcare system’s patient portal to survey patients was also found impracticable, as surveys cannot be targeted to specific clinics. We instead offered paper and telephone patient surveys.

#### D. Implementation climate

##### 2. Compatibility

Mid-level healthcare system managers reported that medical assistants were already too busy with other assigned tasks to take on a larger role in our study. Adding our intervention tasks would be overwhelming and likely not conducted across intervention clinics with acceptable fidelity. Medical assistants also did not have a similar role in the cardiovascular CDS studies, cannot initiate all needed orders in the healthcare system EHR, and roomed with nurses. Consequently, we replaced this intervention arm with an arm that includes the cancer prevention CDS as well as evidence-based SDMT for breast, colorectal, and lung cancer screening and HPV vaccination (child/parent and adult versions) (Table 3). The cancer prevention CDS study now has three arms: a control arm receiving usual care; an intervention arm where PCPs review the cancer prevention CDS with eligible patients; and an intervention arm where PCPs review the cancer prevention CDS with eligible patients along with SDMT. Medical assistants, and other rooming staff, still receive the alert to print the integrated CDS displays in both intervention arms, giving the patient version, along with any relevant SDMT in one intervention arm, directly to eligible patients before PCPs enter the room, and giving the PCP version to the PCP, which is the recommended workflow in the cardiovascular CDS studies. We made this decision based on the movement towards shared decision-making within the healthcare system and evidence that decision aids are helpful tools [4, 13, 14]. We employed a lung cancer SDMT adapted by HealthPartners Institute from the Agency for Healthcare Research and Quality (AHRQ) [37] lung cancer screening clinical decision aids, which follows the latest USPSTF recommendations [33]. We also developed our own SDMT for breast and colorectal cancer screening and HPV vaccination based on the latest USPSTF and Advisory Committee on Immunization Practices (ACIP) recommendations [34, 35, 38], as well as healthcare institution practices due to the exorbitant cost of purchasing existing tools. HealthPartners Institute patient education partners revised these tools to an appropriate reading level, which were then approved by healthcare system patient and family primary care advisory council members.

**Table 3:** Study SDMT Types and Rationale for Development or Use

	SDMT			
Cancer prevention focus:	Breast Cancer	Colorectal Cancer	Lung Cancer	HPV Vaccination
Type of shared decision-making tool:	Team developed based on latest evidence and USPSTF recommendations [34].	Team developed based on latest evidence and USPSTF recommendations [35].	Tool developed by HealthPartners Institute, adapted from the AHRQ lung cancer clinical decision aids [37].	Team developed based on latest evidence and ACIP recommendations [38].
Rationale for development or use:	Too costly for the healthcare system to purchase – Free to develop.	Too costly for the healthcare system to purchase – Free to develop.	Free to use, based on latest evidence and USPSTF recommendations [33].	Too costly for the healthcare system to purchase – Free to develop.

*Note.* ACIP, Advisory Committee on Immunization Practices; AHRQ, Agency for Healthcare Research and Quality; HPV, human papillomavirus; SDMT, shared decision-making tool; USPSTF, U.S. Preventative Services Task Force

#### E. Readiness for implementation

### 2. Available resources

The two cardiovascular CDS studies already underway in the 36 healthcare system clinics influenced our randomization scheme. We are utilizing the same 24 intervention and 12 control clinics, as these clinics had the largest patient populations. We randomized the 24 intervention clinics (including three clinics randomized together due to shared providers) into two balanced clinic groups randomly assigned to receive either the integrated CDS or the integrated CDS plus SDMT. Additionally, we adapted colorectal cancer prevention CDS and SDMT content based on our discussions with healthcare system physicians with subject matter expertise. Flexible sigmoidoscopy and fecal occult blood tests are no longer offered in the healthcare system. Consequently, we removed these options from the colorectal cancer SDMT and CDS. The healthcare system also utilizes Cologuard® DNA fecal immunochemical tests (FIT), which we added to the SDMT and incorporated into CDS ordering. Pilot testing also showed that PCPs can order immunoassay fecal occult blood (IFOB) tests, but patients receive non-DNA FIT tests. Thus, we included IFOB/FIT as a single option in the CDS and colorectal cancer SDMT.

## Discussion

Cancer prevention, screening, and shared decision-making between patients and PCPs may be enhanced by EHR-linked CDS systems and SDMT. Tailored, patient-focused CDS interventions utilizing a team approach may also reduce PCP workload burden and burnout. In this paper, we describe incorporating cancer prevention and screening CDS into an existing cardiovascular CDS that was shown to be effective in previous research [39–42]. We then adapted the cancer prevention and screening CDS to the rural multi-state healthcare system’s goals and culture prior to implementation. We did so through engaging with and gaining feedback from leaders and providers, including by attending primary care medical group and other healthcare system meetings, interviewing key informants [15], and pilot testing the CDS and SDMT. Although we added a new SDMT study arm, medical assistants and other rooming staff still present patients with cancer prevention CDS materials for discussion with their PCP as supported in the literature [17–23]. The healthcare system encourages a team model of patient care, which our revised protocol supports.

CDS systems are often complex, requiring extensive algorithms and coding to correctly capture EHR cancer prevention and screening orders and patient medical history data. With over 50 years of development, CDS still face barriers to use [43]. CDS systems appear to require thoughtful integration into existing clinic workflows in order to be adopted by busy PCPs. In 2003, Bates and colleagues presented the “Ten commandments for Effective Clinical Decision Support” [44], which still hold true today. Speed really is still everything [44], especially in primary care settings. While the goal of the cancer prevention CDS is to anticipate patients’ cancer prevention and screening needs and deliver them to patients and providers in real time, interventions like the cancer prevention CDS will only be adopted and used if it fits into exiting workflows without hard stops and if it is user-friendly [44]. Our key informants emphasized the importance of each of these areas [15], as well as the need for continual monitoring and maintenance of CDS performance [44], which we attempted to address in the CDS design. However, although we conducted a 6-month silent pilot in one non-study healthcare system clinic and a 7-week live pilot in two additional non-study clinics, we discovered post-implementation that neither were long enough to capture or correct all potential issues. The site Principal Investigator, project manager, and research coordinators traveled to all 26 intervention arm clinics to provide in-person, on-site CDS training for clinic rooming staff, PCPs, and management. Additional adaptations were made based on clinic feedback received after going live with the intervention and included developing and implementing half page SDMT that automatically print with the patient and provider CDS handouts. This was primarily due to the length of the full length SDMT, which range from 2 to 4 pages, and the amount of paper that was being printed. Full length SDMT are still available for printing within the CDS interface in the EHR. We also uncovered and addressed multiple printer issues, some related to clinic computers not being correctly mapped to network printers, driver issues, and/or printer firmware. However, unidentified printing problems continue to be an issue for some clinics. Furthermore, to encourage usage, we instituted an incentive program for clinics that consistently have high CDS utilization. Since the cancer prevention CDS intervention went live in June 2018, we have also continued to engage with our primary care clinics and healthcare system leaders. The study is currently in a 12-month follow-up period, after which full study results will be available. We are continuing to monitor CDS use, troubleshoot technical issues as they arise, and collect data from patients, PCPs, rooming staff, and clinic leaders to further inform our ongoing D&I efforts.

### Transportable lessons

Based on our experiences, we can share seven transportable lessons for other researchers modifying current CDS systems pre-implementation.

#### 1. One size does not fit all

We recommend following models of CDS that have proven track records for success, while being prepared to adapt to unique site characteristics prior to implementation. A CDS system that works well in one healthcare system or setting may not translate into another. Although the cancer prevention CDS was integrated into an existing and successful cardiovascular CDS that was based in urban settings [39–42], we had to make adaptations for it to function most effectively with new clinical domains and in a new healthcare system setting with a more rural population and different clinic resources. Moreover, the team had to adapt the implementation strategy given each clinic’s unique culture around cancer prevention, the use of the EHR to address cancer prevention, as well as overall clinic interest in using the integrated CDS.

#### 2. Use a guiding framework – or more than one

The research team selected the CFIR as one of our guiding D&I frameworks in the grant writing phrase and used it when conducting our key informant interviews [15]. However, since that time, Van de Velde and colleagues published the GUIDES checklist that is specifically focused on helping CDS developers create more successful CDS [45]. Medlock and colleagues also proposed a “two-stream model” that CDS developers can use as an additional checklist for identifying potentially influential barriers and facilitators to CDS effectiveness [46]. Greene and colleagues recently outlined a number of additional models and frameworks related to CDS, noting that more than one may be needed due to the complexity of CDS [44].

#### 3. Gain front-line key informant input early – and sustain those relationships

Early feedback from front-line CDS users, such as clinic rooming staff and PCPs, may be most helpful in designing a CDS intervention that works best with or can adapt to clinic workflow. Plan on sustaining those relationships while continuing to identify and adapt to healthcare system concerns after implementation through routine solicitation of feedback. Doing so could not only continually improve the intervention, but also make it easier to undertake future pragmatic trials within the healthcare system.

#### 4. CFIR domains and constructs may overlap or not be applicable pre-implementation

Although the CFIR was designed to eliminate redundancies between other implementation frameworks [26], we found that multiple CFIR domains and constructs could apply to a single change. We encourage others to interweave CFIR domains and constructs as needed to more completely describe changes made [26]. Also, not all CFIR domains or constructs may be appropriate pre-implementation. Other models, frameworks, or checklists more specific to CDS [44], like the GUIDES checklist or “two-stream model” [45, 46], could also be applied during CDS development in addition to the CFIR.

#### 5. Do not underestimate the challenges of technology

Algorithms working between two organizations using different versions of the same EHR, including differing codes, made it difficult to programmatically search EHR reports and scanned documents. We engaged study healthcare system information support staff during CDS development and implementation to troubleshoot these issues as they arose. As we noted, we experience multiple issues with printers used by primary care clinics for printing patient materials. The research team has continued working on printing issues during the intervention and follow-up periods, troubleshooting problems as they arise.

#### 6. Start small

Prior to implementation, we anticipated that the CDS would print at a higher frequency than the cardiovascular CDS and that doing so would drive utilization down – it did at first. Big changes within an existing intervention may be best accomplished with small stepwise changes over a longer period of time rather than all at once.

#### 7. Prepare for continual adaptation

Do not be afraid to continue modifying your intervention if it is apparent that there are significant issues in the workflow across many intervention clinics. The nature of pragmatic CDS studies across multiple primary care clinics within multiple states requires adaptability. Clinic workflows will differ, CDS recommendations will change, technology will advance, and you must be prepared to take seriously the feedback you receive from PCPs, rooming staff, and leadership. Modifications will be required if the same feedback is received over an extended period of time.

### Limitations

This study did have limitations. This paper only focuses on pre-implementation changes made based on barriers and facilitators identified from the perspective of healthcare system leaders, providers, PCPs, medical assistants, and other rooming staff. Additional post-implementation D&I efforts are planned with these individuals, as well as with patients. However, study team members carefully reviewed the intervention and protocol changes presented here for accuracy. We also reported separately the results from our key informant interviews, which led to some of the changes described here [15]. Another limitation is that we report no statistical results in this paper, as quantitative data were unavailable on the cancer prevention CDS prior to full implementation. However, the primary aims of the overarching randomized control trial include evaluating the effectiveness of the cancer prevention CDS, the results of which will be published at the end of the trial.

## Conclusions

EHR-based decision aids may improve cancer prevention and screening in primary care. We employed the CFIR [26] in describing pre-implementation changes made based on barriers and facilitators encountered when incorporating cancer prevention and screening into a current cardiovascular risk management CDS system to facilitate implementation and maximize use of the integrated CDS system. We also provide a number of transportable lessons on altering existing CDS with new clinical domains and/or implementing CDS into new settings. Other researchers may benefit from these lessons when undertaking similar efforts of updating current CDS system interventions with new clinical aims and implementing them in new settings. Given the nature of this pragmatic research study conducted within a dynamic healthcare system spread across a diverse and rural region, the research team has had to continually tailor their implementation and adaptation approach, as well as customize strategies based on clinic culture. While the randomized control trial is still underway, the research team continues to actively engage with the intervention clinics’ PCPs, managers, and rooming staff. Future research is planned to understand best approaches to system-wide dissemination of the CDS, as well as promote continued high usage among primary care clinic rooming staff and PCPs.

## Data Availability

The datasets used and/or analyzed during the current study are available from the corresponding author on reasonable request.

## Abbreviations

ACIP: Advisory Committee on Immunization Practices
AHRQ: Agency for Healthcare Research and Quality
CDS: Clinical decision support
CFIR: Consolidated Framework for Implementation Research
D&I: Dissemination and implementation
EHR: Electronic health record
FIT/FIT DNA: Fecal immunochemical test / Fecal immunochemical test deoxyribonucleic acid
HPV: Human papillomavirus
IFOB: Immunoassay fecal occult blood test
PCP: Primary care provider
USPSTF: United States Preventative Services Task Force
